# De Novo Atypical Haemolytic Uremic Syndrome after Kidney Transplantation

**DOI:** 10.1155/2018/1727986

**Published:** 2018-03-14

**Authors:** Arnaud Devresse, Martine de Meyer, Selda Aydin, Karin Dahan, Nada Kanaan

**Affiliations:** ^1^Division of Nephrology, Cliniques Universitaires Saint-Luc, Université Catholique de Louvain, Brussels, Belgium; ^2^Division of Abdominal Surgery and Transplantation, Cliniques Universitaires Saint-Luc, Université Catholique de Louvain, Brussels, Belgium; ^3^Division of Pathology, Cliniques Universitaires Saint-Luc, Université Catholique de Louvain, Brussels, Belgium; ^4^Division of Human Genetics, Cliniques Universitaires Saint-Luc, Université Catholique de Louvain, Brussels, Belgium; ^5^Center of Human Genetics, Institut de Pathologie et de Génétique, Gosselies, Belgium

## Abstract

De novo thrombotic microangiopathy (TMA) can occur after kidney transplantation. An abnormality of the alternative pathway of complement must be suspected and searched for, even in presence of a secondary cause. We report the case of a 23-year-old female patient who was transplanted with a kidney from her mother for end-stage renal disease secondary to Hinman syndrome. Early after transplantation, she presented with 2 episodes of severe pyelonephritis, associated with acute kidney dysfunction and biological and histological features of TMA. Investigations of the alternative pathway of the complement system revealed atypical haemolytic uremic syndrome secondary to complement factor I mutation, associated with mutations in CD46 and complement factor H related protein genes. Plasma exchanges followed by eculizumab injections allowed improvement of kidney function without, however, normalization of creatinine.

## 1. Background

De novo thrombotic microangiopathy (TMA) has been reported to occur after kidney transplantation [1]. The pathogenic mechanisms are not well understood but are likely multifactorial with implication of specific features attributed to kidney transplantation [2]. However, the implication of a dysregulation in the alternative complement pathway may be underestimated [3].

## 2. Case Report

**Figure 1:**
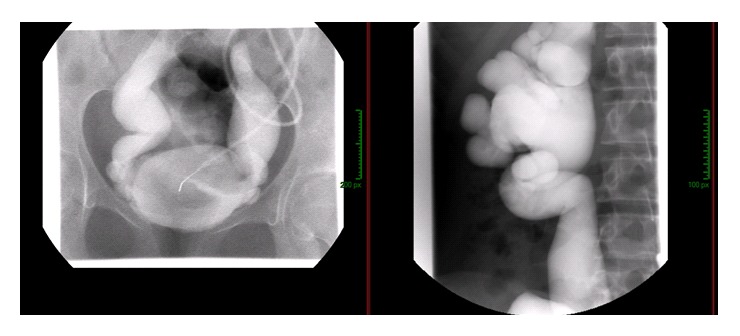
Retrograde cystography showing massive ureterovesical reflux and hydronephrosis.

A 14-year-old female patient was admitted 9 years ago to our institution for acute pyelonephritis. Massive bilateral ureterohydronephrosis secondary to grade V ureterovesical reflux associated with a trabeculated bladder was evidenced (Figure 1). Neurologic investigations revealed no abnormality, leading to a diagnosis of Hinman syndrome, a very rare entity characterized by all features of a neurogenic bladder with external sphincter dyssynergia, but without evidence of any neurologic alteration [4]. After diagnosis, she had several uncomplicated urinary tract infections, necessitating self-catheterization (importantly never associated with biological thrombocytopenia or haemolytic anemia), and reached end-stage renal disease at age 23 when she underwent preemptive HLA semi-identical living-donor (her mother) kidney transplantation.

**Figure 2:**
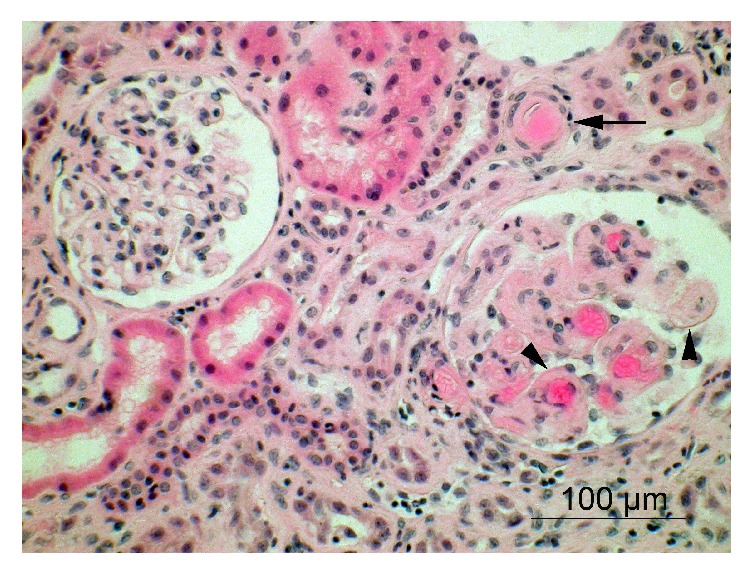
Histological examination showing thrombotic microangiopathy in a kidney biopsy from renal allograft at day 120 (hematoxylin and eosin). Microthrombi and lucent deposits (arrowheads) are observed in the glomerulus at the right side, with obstruction of a nearby arteriole by eosinophilic material (arrow). Notice the unaffected glomerulus on the left side of the microphotograph. There is no evidence of acute antibody-mediated rejection according to the 2015 Banff classification (g0, ptc0, and no C4d deposit by immunofluorescence (not shown)).

Her immunosuppressive regimen included Basiliximab induction, tacrolimus, mycophenolate mofetil, and steroids. She was discharged at day 11 with a normal plasma creatinine (1.26 mg/dl). At day 40, she was admitted for intestinal occlusion due to adhesions requiring adhesiolysis. During hospitalization, she presented a severe pyelonephritis secondary to* Pseudomonas aeruginosa* (colony count > 100,000 colony-forming units of bacteria per mL of urine with negative blood cultures but with increased level of C-reactive protein, features consistent with acute pyelonephritis), associated with acute renal failure (creatinine 2.73 mg/dl), increased lactate dehydrogenase level, decreased haptoglobin, haemoglobin, and platelets level. Tacrolimus trough levels were elevated. Complement 3 and 4 levels were normal. Donor-specific antibodies (DSA) were negative (Table 1). She was treated with antibiotics, and a kidney biopsy performed 48 hours later was normal with no sign of acute rejection, acute pyelonephritis, or acute tubular necrosis. Interstitial fibrosis and tubular atrophy (IFTA) was scored 1. Because of the severity of the biological signs of thrombotic microangiopathy (TMA), she was treated with daily plasma exchanges with fresh frozen plasma for one week. Laboratory tests normalized except creatinine that remained elevated (2.1 mg/dl). At day 120, the patient was admitted again for severe pyelonephritis secondary to* Pseudomonas aeruginosa *(>100,000 colony-forming units of bacteria per mL of urine with negative blood cultures) with acute renal failure (creatinine 5.5 mg/dl) and the same biological picture (Table 1). A second kidney biopsy showed pathognomonic features of thrombotic microangiopathy (including a preglomerular arteriole of one glomerulus obstructed by a fresh thrombus and mesangiolysis, without argument for antibody-mediated rejection) (Figure 2). IFTA was scored 1. DSA were absent. Screening for secondary causes of TMA was negative (antiphospholipid syndrome, Shiga toxin, ADMATS13 deficiency or inhibitor, antinuclear factor, Coombs test, disseminated intravascular coagulation, HIV, CMV, pregnancy, hypertension, or occult infection). A genetic screening of the alternative complement pathway revealed a heterozygous mutation in complement factor I* (CFI)* gene c.148C>G (p.(Pro50AIa)), associated with a heterozygous variant of membrane cofactor protein* CD46* (c-366A) and a homozygote deletion of complement factor H* (CFH)* related protein* CFHR1 *and* CFHR3 *genes. Anti-FH antibody screening was negative. The patient received two plasma exchanges with fresh frozen plasma while waiting to have fast access to anti-C5 antibody. Eculizumab was then started using the recommended doses (900 mg/week for 1 month and then 1200 mg/2 weeks). She was treated for 10 months (the period allowed by our legislation).

Creatinine level stabilized around 2 mg/dl. An allograft biopsy performed 3 months after initiation of eculizumab showed no sign of TMA but a progression of IFTA score to 2. She did not experience any infectious event or biological signs of TMA under eculizumab and is currently doing fine 3 months after treatment cessation.

Screening of the mother revealed the same mutations in* CFI* and* CFHR1–CFHR3*. Her pre-kidney-donation work-up was normal and the postnephrectomy evolution was uneventful. Genetic screening was proposed to other members of family (patient's father and brother) but has not been performed yet.

## 3. Discussion

TMA is a pathologic description, clinically characterized by an association of thrombocytopenia, microangiopathic haemolytic anemia and organ injury [1]. After solid organ transplantation (including kidney, liver, pancreas, lung, and heart) or bone marrow transplantation, de novo TMA had been reported to occur [1, 5, 6]. In the kidney transplantation setting, de novo TMA classically occurred in the 6 first months after kidney transplantation [7] with an incidence between 0.8% to 14% [3, 7]. If the pathogenic mechanisms of de novo TMA are not well understood, they are likely to be multifactorial with ischemia-reperfusion injury, antibody-mediated rejection, viral infection such as cytomegalovirus and immunosuppressant drugs, especially calcineurin inhibitors (CNI), contributing to an “endothelial damaging milieu” [2]. In many cases, supportive treatment and addressing the precipitating factors (CNI dose reduction, CNI withdrawal, treatment of acute antibody-mediated rejection, and viral infections) are sufficient to stop TMA [1]. However, for some patients, this strategy does not lead to an improvement of TMA. For those patients, a complement-mediated TMA secondary to a dysregulation of the alternative complement pathway, classically called atypical haemolytic and uremic syndrome (a-HUS), should be suspected. a-HUS is a rare disorder due to genetic mutation of the alternative complement pathway [8]. These mutations can be found in the regulatory genes* (CFH, CD46, CFI, Thrombomodulin)* or in the activatory genes (*factor B*,* C3*). a-HUS can also be secondary to anti-CFH antibodies [8]. A trigger event such as infection or pregnancy is believed to precipitate a-HUS in a susceptible individual. Making the genetic diagnosis of a-HUS before kidney transplantation is crucial: first, the risk of recurrence after kidney transplantation depends on whether the mutant complement factor is membrane-bound (low risk) or circulating (high risk) [9]; second, the introduction of eculizumab, a terminal complement inhibitor, as preventive treatment, has dramatically improved the risk of a-HUS recurrence after kidney transplantation leading to a huge improvement in the allograft survival in these patients [10–12].

In the setting of de novo TMA after kidney transplantation, the implication of a dysregulation in the alternative complement pathway may be underestimated as suggested by one series of de novo TMA after kidney transplantation published by le Quintrec et al. [3]. In a cohort of 24 deceased-donor kidney transplant recipients who experienced de novo TMA after kidney transplantation and who had systematic screening for mutations in genes encoding CFH, CFI, and CD46, 7/24 patients were found to have a mutation: 1 CFH, 4 CFI, and 2 CFH and CFI. Mutations in* CFI* are heterozygous in most patients. Interestingly, 30% of patients with* CFI* mutation were found to have an additional mutation in genes known to be susceptible risk factors for a-HUS. The diagnosis of a-HUS in our patient before transplantation was not suspected. Indeed, she did not show any signs of TMA despite several episodes of urinary tract infections. Her mother also never experienced signs of TMA neither before transplantation (despite 2 pregnancies) nor after kidney donation. The genetics of these mutations is highly complex with a penetrance around 50% [13]. Familial studies suggest a monoallelic autosomal or pseudoautosomal mode of inheritance [14, 15]. Moreover, within families, affected persons may also show different symptoms and ages at onset of the disease [13]. This highly suggests that most a-HUS-associated genetic variants predispose to rather than cause the disease and that triggers are necessary to develop symptoms as for our patient who exacerbated a-HUS symptoms only in the presence of several pathologic conditions: kidney transplantation, immunosuppression, and infection.

Our patient improved her allograft renal function after eculizumab initiation suggesting that, besides preventing recurrence after transplantation, it can be efficient to reverse the fate of renal function in de novo a-HUS occurring after kidney transplantation as demonstrated in a-HUS occurring in the nontransplant setting [11]. Treatment duration of eculizumab is controversial. Despite early recommendations for a lifelong therapy, there is no evidence supporting this attitude [16]. Recently, reports have suggested that, in native kidneys, eculizumab therapy may be discontinued after remission has been achieved, with a prompt resumption of therapy in cases of relapse. Wijnsma et al. reported 20 non-kidney-transplant patients, in whom a restrictive treatment in time followed by a TMA monitoring appeared safe and effective [17]. In kidney transplant recipients, Duineveld et al. reported recently a case series including 17 patients with a-HUS who underwent living kidney transplantation without prophylactic eculizumab. A monitoring strategy was applied and was successful, as only one patient experienced recurrence, which was successfully treated [18]. The good outcomes in this report may be due to the fact that (1) all living donors were genotyped, (2) cold ischemia time was short, and (3) low targets of tacrolimus were used [19]. In our patient, eculizumab was discontinued after 10 months due to limitations imposed by our national reimbursement policy. Currently, 3 months after therapy cessation, she is doing fine with a monthly biological screening. Prospective studies including larger cohorts of kidney transplant recipients with a long follow-up are required to assess whether eculizumab prophylaxis should be restricted to specific profiles and to assess treatment duration.

In conclusion, our case highlights the importance of the following: (1) a genetic screening in de novo TMA after kidney transplantation, (2) identifying the underlying mutation allowing treatment that can potentially reverse the fate of renal function, (3) familial screening and counselling in the context of living donation in case of suspected biological TMA in the donor and/or the recipient.

## Figures and Tables

**Table 1:** Laboratory findings.

	Day 11°	Day 40°	Day 120°
C-reactive protein, mg/L (*N* < 5.0)	33.0	117.0	452.0
Plasma creatinine, mg/dL (*N*: 0.60–1.30)	1.21	2.73	5.5
Lactate dehydrogenase, IU/L (*N* < 250)	376	722	517
Hemoglobin, g/dL (*N*: 12.2–15.0)	9.6	7.7	7.0
Coombs test	NA	Negative	Negative
Platelets count, per *μ*/L (*N*: 150000–450000)	417,000	51,000	96,000
Haptoglobin, g/L (*N*: 0.3–2.0)	NA	<0.1	<0.1
Schistocytes count, % of red blood cells	NA	4	2
Tacrolimus trough level, ng/mL	9.0	26.5	9.9
Anti-HLA antibody screening^*∗*^	NA	Negative	Negative
Complement C4, g/L (*N*: 0.1–0.4)	NA	0.34	0.36
Complement C3, g/L (*N*: 0.9–1.8)	NA	1.14	1.53
CMV (PCR), copies/mL	Undetected	Undetected	Undetected

°After kidney transplantation. ^*∗*^Class I and class II anti-HLA antibody screening performed by single antigen bead assay. CMV: cytomegalovirus; HLA: human leukocyte antigen; IU: international unit; PCR: polymerase chain reaction; *N*: normal value; NA: not available.
